# Validation of the Asian Midface Volume Deficit Severity Scale

**DOI:** 10.1007/s00266-025-04723-0

**Published:** 2025-06-25

**Authors:** Jingyu Li, Lei Pan, Qing Yun, Jinyu Xia, Linna Wang, Xiaodong Shen, Shaoli Chi, Hongxia Zou, Yanyan Zhao, Sufan Wu

**Affiliations:** 1 Center for Plastic and Reconstructive Surgery, Department of Plastic and Reconstructive Surgery, Zhejiang Provincial People’s Hospital(Affiliated People’s Hospital), Hangzhou Medical College, 158# Shangtang Road, Hangzhou, Zhejiang China; 2 Air Force Medical Center, PLA, Beijing, China; 3 Peking University First Hospital, Beijing, China; 4 Lanzhou Biotechnique Development Co, Ltd, Gansu, China; 5 China National Biotec Group Company Limited, Beijing, China; 6 Medical Research and Biometrics Center, National Center for Cardiovascular Diseases, Cardiovascular Institute and Fuwai Hospital, The Chinese Academy of Medical Sciences, Beijing, China

**Keywords:** Aesthetics, Midface volume, Asian, Dermal fillers, Validation studies

## Abstract

**Background:**

Anatomical differences in facial aging and aesthetic needs of Asian and Caucasian individuals have led to disparate clinical rejuvenation strategies using filler augmentation for midface volume deficits. Therefore, the Asian Midface Volume Deficit Severity Scale (AMVDSS) by LBTD (Lanzhou Biotechnique Development Co, Ltd) was developed to objectively assess midface volume deficits and clinical outcomes of Asian individuals.

**Methods:**

Four observers independently rated randomized photographs (front, left and right 45°, left and right 90°) of 65 patients during two sessions with a 2-week interval using the AMVDSS.

**Results:**

All participants were Asian (18 male and 47 female participants; mean age, 40.9 years; range, 20–76 years). Interobserver agreement was substantial to almost perfect for the left (weighted κ, 0.778–0.907) and right (weighted κ, 0.774–0.938) during the first session; it was almost perfect for the left (weighted κ, 0.844–0.907) and right (weighted κ, 0.805–0.876) during the second session. Interobserver agreement was 76.9% to 90.8% and 76.9% to 93.9% for the left and right, respectively, during the first session; it was 84.6% to 90.8% and 81.5% to 87.7% for the left and right, respectively, during the second session. Intraobserver agreement was “almost perfect” for both the left (weighted κ, 0.950; 95% confidence interval [CI] 0.923–0.977) and right (weighted κ, 0.954; 95% CI 0.928–0.980). Exact intraobserver agreement was 95.0% (range, 90.8–100.0%) for the left; it was 95.4% (range, 94.9–96.9%) for the right.

**Conclusions:**

The AMVDSS by LBTD could be used to reliably assess midface volume deficits or volume changes after dermal filler injections.

**Level of Evidence II:**

This journal requires that authors assign a level of evidence to each article. For a full description of these Evidence-Based Medicine ratings, please refer to the Table of Contents or the online Instructions to Authors www.springer.com/00266

## Introduction

Facial aging is a multidimensional and multifactorial process involving all facial layers and includes skull shape changes, fat loss, soft tissue ptosis, and reduced skin elasticity. [1] Aging of the midface is caused by ligament relaxation, and soft tissue ptosis leads to the appearance of midface volume deficits. For Asian individuals, facial soft tissue ptosis is the primary marker of aging instead of wrinkles. [2] Midface augmentation using fillers has become a common choice for skin rejuvenation. Therefore, physicians and researchers require a validated scale to objectively assess clinical outcomes of midface rejuvenation.

Because soft tissue fillers have a long history, researchers have attempted to develop reliable midface scales. Several validated midface scales have been used for clinical research. However, scales for the midface, unlike other regions of the face such as forehead rhytids or glabellar lines, require more research. The midface is a dimensionally complicated region of the human face that is difficult to precisely describe. Commonly used midface scales include the 6-point midface volume deficit scale (MFVDS) and the 4-point Medicis midface volume scale (MMVS). [3, 4] Descriptions of the different severity levels of the MFVDS are broad and complicated and include the tear troughs, nasolabial folds, zygomaticomalar region, anteromedial cheek, and submalar region. [5, 6] Descriptions of the different severity levels of the MMVS include the nasojugal groove, submalar region, and total fullness of the midface area [7, 8].

The facial anatomy and aesthetic needs of Asian and Caucasian individuals are quite different. Although the MFVDS has been validated for the Asian population, the differences in clinical strategies for midface augmentation of Asian individuals cannot be ignored. Midface augmentation using fillers such as hyaluronic acid for Caucasian individuals usually involves injections in the zygomaticomalar region, anteromedial cheek, and submalar region. [9, 10] However, the aim of midface rejuvenation for Asian individuals is to project the medial cheeks without widening the malar. [11] Therefore, Asian individuals commonly choose to receive filler injections only in the anteromedial cheek because they want to fill the defect in this region and look younger and fresher without exaggerated changes of the face. [12] Scoring with the already validated MFVDS or MMVS is difficult when only the anteromedial cheek region has been filled with unchanged nasojugal groove and submalar region, and has been an unresolved problem in the clinical practice among Asian people. The validated midface scales of the anteromedial cheek and submalar region require improvement. Therefore, another midface volume scale was developed to objectively assess the midface volume deficits and clinical outcomes of Asian individuals.

The 4-point Asian MVDSS (AMVDSS) by LBTD was developed to measure the midface volume deficit of Asian individuals (Table 1). It is the first scale for the clinical assessment of midface fullness of Asian individuals. The most important difference between this scale and the scales developed mainly for Caucasian people is that AMVDSS only evaluates the anteromedial cheek region. This photographic scale is used to grade the midface as “nearly full or slight loss of fullness” (grade 1) to “severe loss of fullness” (grade 4). This study evaluated the intraobserver agreement and interobserver agreement for the AMVDSS by LBTD (i.e., each observer could reproduce his or her original grade at different times and the degree to which different observers independently gave an identical score to the same subject, respectively).

**Table 1:** Description of the Asian midface volume deficit severity scale

Grade	Definition
1	Nearly full or slight loss of fullness of the midface
2	Mild loss of fullness of the midface, presence of a grooving deformity without broadening at the midline of the pupil
3	Moderate loss of fullness of the midface, presence of a grooving deformity with concavity extending across the midline of the pupil and broadening to form a regional depression
4	Severe loss of fullness of the midface, presence of a grooving deformity with concavity extending across midline of pupil and broadening to form a severe regional conical depression

## Materials and Methods

### Participants and Selection of Photographs

This validation study included 65 participants. All participants provided written informed consent to participate in this study and for use of their photographs. All photographs were obtained in a single room with the same light conditions using the same camera (Canon EOS R7). At least two photographs of each facial angle (front, left and right 45°, left and right 90°) were obtained. Five photographs (one photograph of each angle) of each participant were chosen to show the full view of the face and for scale validation. The photographs of 65 participants were randomized and rated by four independent observers (two plastic surgeons and two dermatologists) who were not involved with scale development but had been trained to use the photographic scale. All observers had more than three year of experiences as filler injectors. The training for the observers included detailed explanation of the scale, discussion-based learning of sample photos, and simulation of scoring. This study was approved by the institutional review board.

### Validation Procedure

**Fig. 1:**
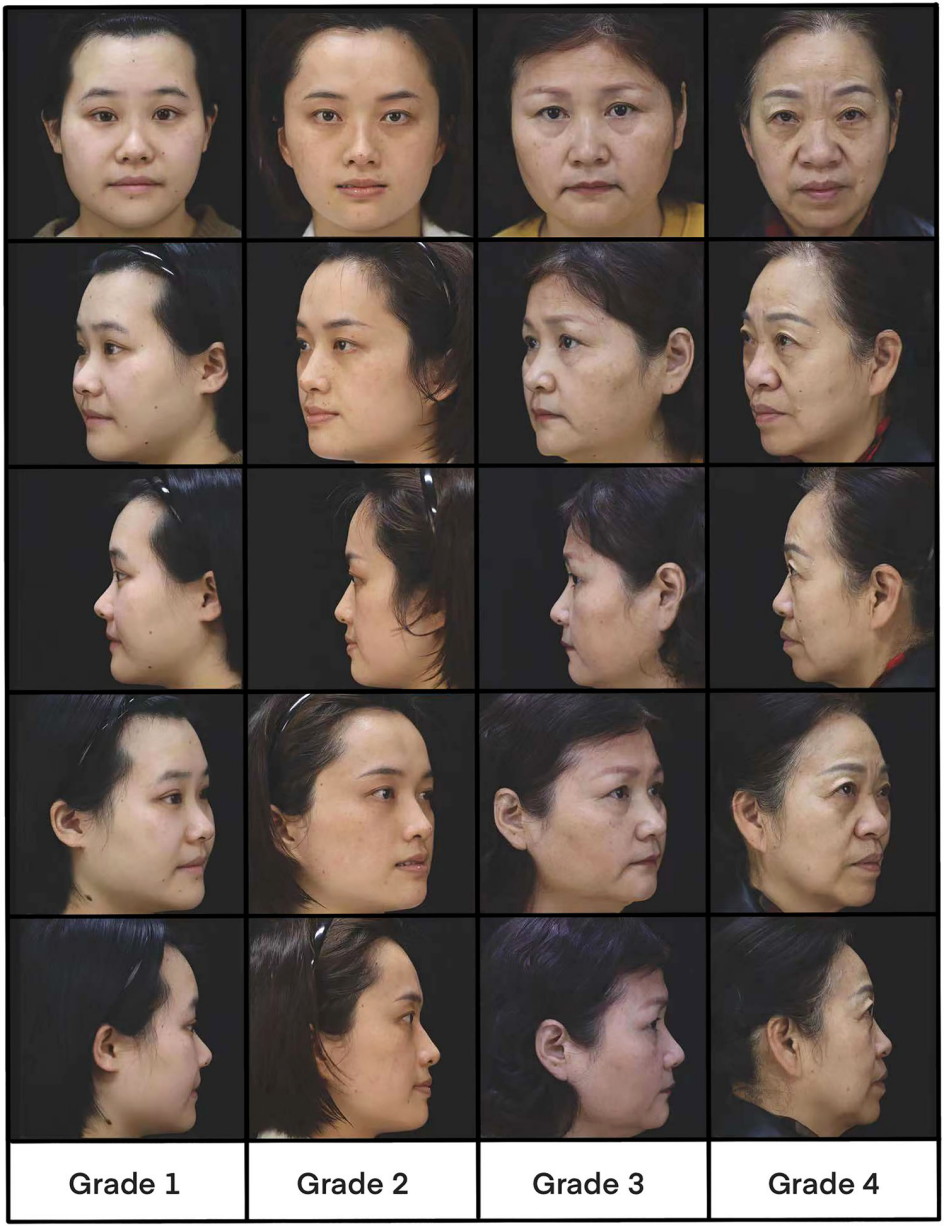
Photographic images used with the Asian Midface Volume Deficit Severity Scale (AMVDSS) by LBTD

Each observer rated each participant’s right and left midface using the AMVDSS by LBTD (Table 1). Each grade of the AMVDSS was exemplified by photographic images (Fig. 1).

### Statistical Analysis

During this study, four observers used the same scale to rate the same participants, and the agreement, weighted κ coefficient, and 95% confidence interval (CI) were used to evaluate the consistency of different raters who evaluated the same participants.[13] The κ coefficient and corresponding 95% CI were used to assess interobserver consistency. The κ coefficients were interpreted as follows: 0 to 0.19, poor agreement; 0.20 to 0.39, fair agreement; 0.40 to 0.59, moderate agreement; 0.60 to 0.79, substantial agreement; and 0.80 to 1.0, almost perfect agreement.

After a 2-week interval, each observer used the same scale to rate the photographs of the same participants (newly randomized) again. The agreement rate, weighted κ coefficient, and 95% CI were used to evaluate intraobserver consistency. The κ coefficient and corresponding 95% CI were used to assess interobserver consistency.

## Results

A total of 65 sets of photographs (five photographs per participant) were used to validate the AMVDSS by LBTD. The mean age of the participants was 40.9±13.8 years (range, 20–76 years). All participants were Asian (18 male and 47 female participants).

Interobserver reliability (i.e., consistency of scores given by different observers) for the AMVDSS during session 1 is shown in Table 2. The agreement of the four observers working together as six pairs (observers 1 and 2, observers 1 and 3, observers 1 and 4, observers 2 and 3, observers 2 and 4, observers 3 and 4) ranged from 76.9 to 90.8% and 76.9 to 93.9% for the left and right sides, respectively, of the 65 participants. Weighted κ values ranged from 0.778 to 0.907 and 0.774 to 0.938 for the left and right sides, respectively, indicating substantial to almost perfect interobserver agreement for the assessment of the midface volume during session 1.

**Table 2:**
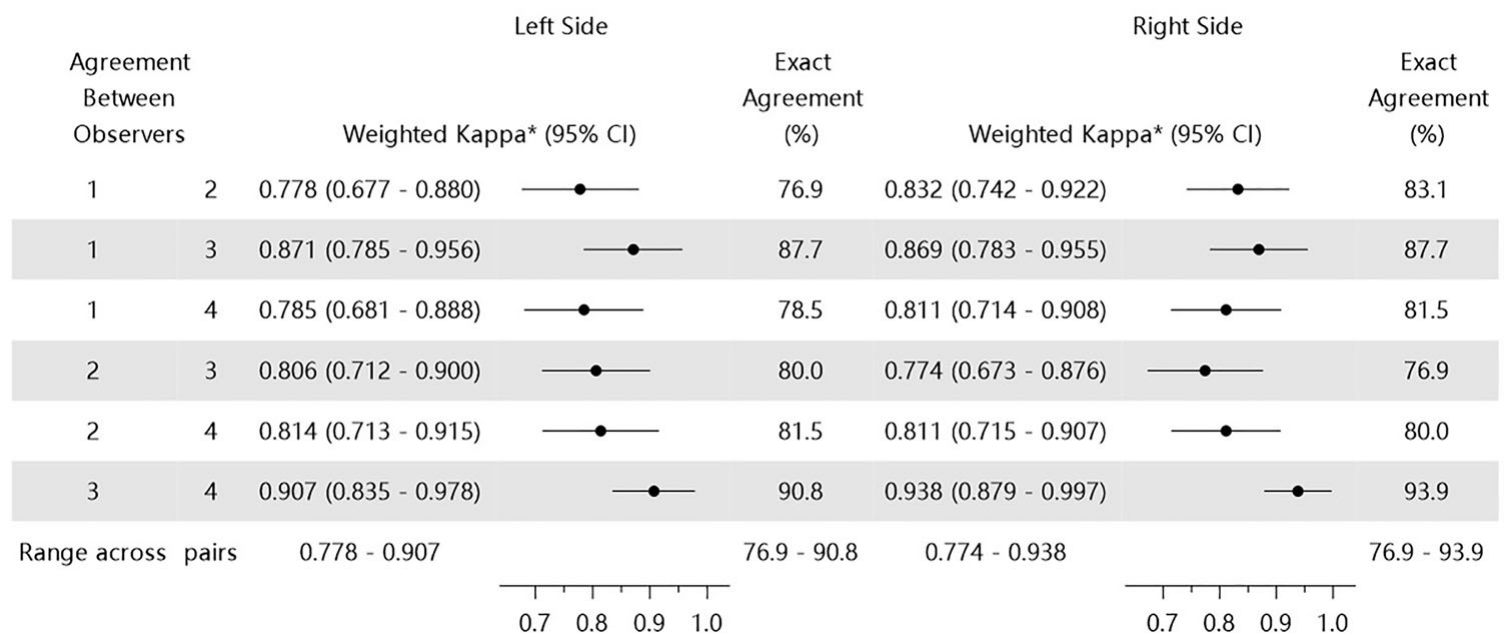
Interobserver reliability of Asian midface volume deficit severity scale grades for the first round

Interobserver reliability (i.e., consistency of scores given by the same observer at different times) for the AMVDSS during session 2 is shown in Table 3. The agreement of the four observers ranged from 84.6 to 90.8% and 81.5 to 87.7% for the left and right sides, respectively. Weighted κ values ranged from 0.844 to 0.907 and 0.805 to 0.876 for the left and right sides, respectively, indicating almost perfect interobserver agreement for the assessment of the midface volume during session 2.

**Table 3:**
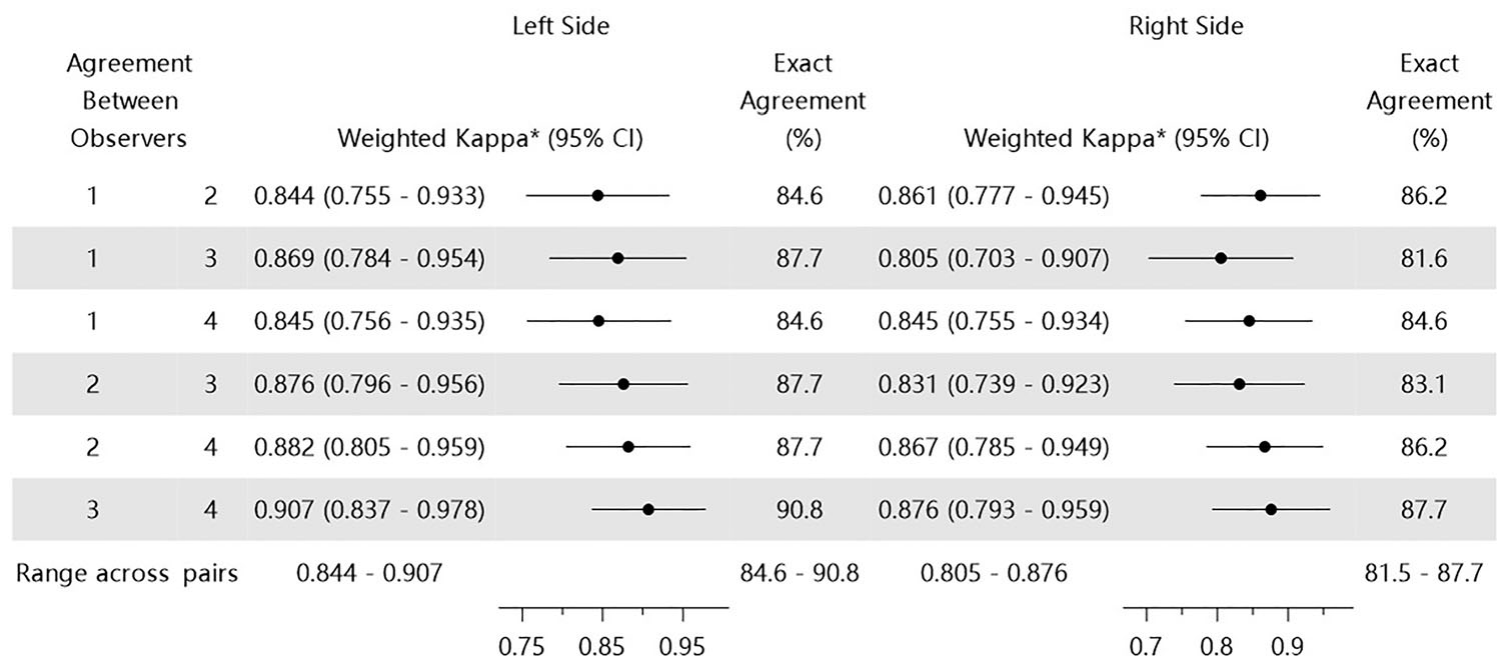
Interobserver reliability of Asian midface volume deficit severity scale grades for the second round

Intraobserver reliability during session 1 and that during 2 are shown in Table 4. Exact interobserver agreement was 95.0% (range, 90.8–100.0%) for the left side and 95.4% (range, 94.9–96.9%) for the right side. The weighted κ coefficients of the four observers were 0.950 (95% CI, 0.923–0.977) for the left side of the face and 0.954 (95% CI, 0.928–0.980) for the right side of the face, indicating almost perfect intraobserver agreement between sessions 1 and 2.

**Table 4:**
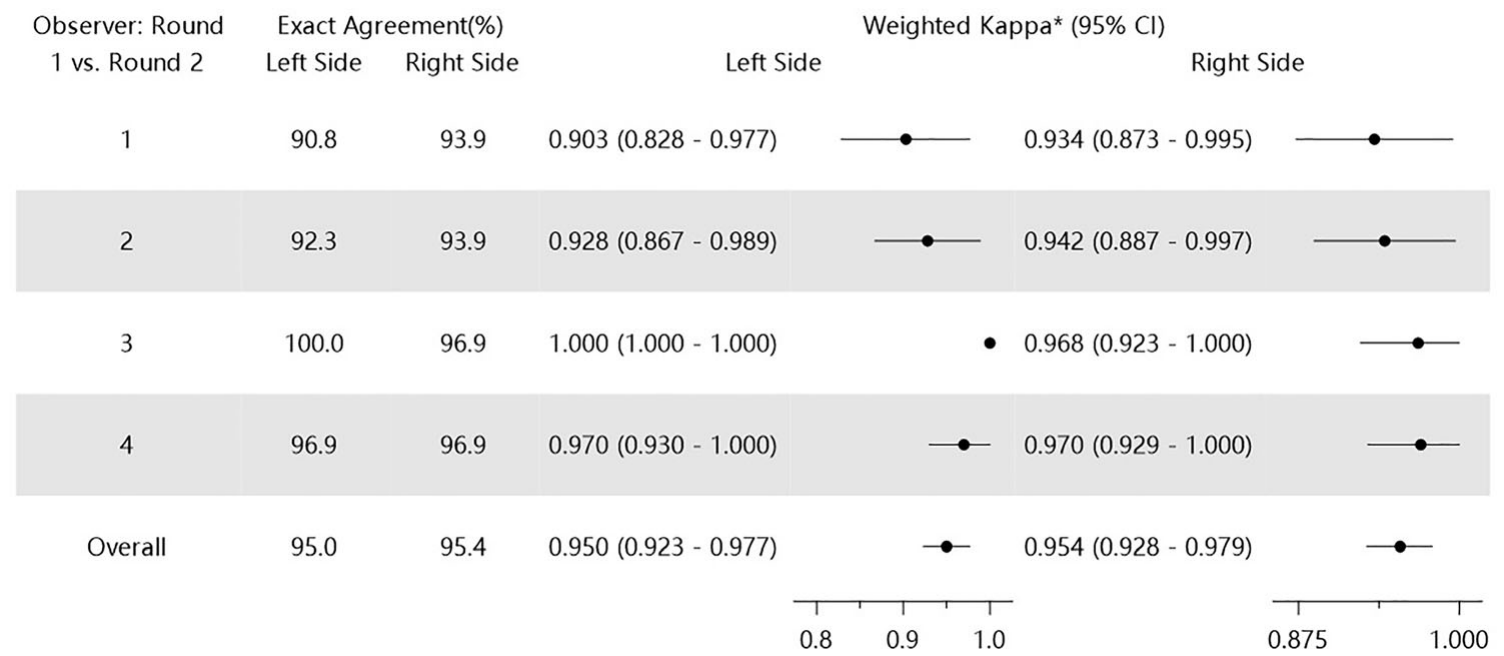
Intraobserver reliability of Asian midface volume deficit severity scale grades

## Discussion

The results of this study demonstrate the validity of the 4-point AMVDSS by LBTD for the objective evaluation of the midface volume among a cohort of Asian adult participants. The interval time (2 weeks) between the two sessions and the randomizing of photographs were implemented to eliminate the memory bias of the observers. The statistical analysis evaluated the consistency of four independent observers. The results showed that both intraobserver consistency and interobserver consistency were high; high consistency was also confirmed by weighted κ statistics.

The results of this study validated the definitions and descriptions of the AMVDSS, and they indicated that the photographs could be exact and reliable in clinical practice. Therefore, the AMVDSS can provide reliable assessments regardless of whether it is used by different evaluators or the same evaluator on different occasions. It could also be used during clinical trials to provide objective assessment results and has potential for application in clinical practice. Furthermore, the AMVDSS can help physicians when assessing midface volume deficits, the need for filler injections, and the efficacy and maintenance time for patients. Additionally, the results of the left and right sides were almost the same, thus indicating that the AMVDSS is reliable when used for either side of the face.

It is noteworthy that interobserver agreement between the first and second sessions showed an increasing trend, which suggested that more thorough training with the scale (the training was reviewed before the second round of scoring) and increased user familiarity can improve the reliability of the scale.

This study had some limitations. First, the limited number of the participants and observers might be associated with potential biases. Future studies may include other ethnic groups to improve the reliability of the scale. Moreover, a larger number of observers, and inclusion of less-experienced or even non-experienced clinicians, might lead to different findings for the reproducibility of the scores. Second, the participants in this study did not undergo augmentation of the midface. Additional research for confirming the applicability of the AMVDSS to assessing clinical outcomes after midface augmentation with hyaluronic acid is in progress. Furthermore, studies involving other fillers may also be necessary to investigate whether different fillers affect the rating results differently.

## Conclusion

Based on the interpretation of the weighted κ coefficients, the intraobserver agreement and interobserver agreement support the applicability of the AMVDSS by LBTD for clinical trials to objectively assess midface volume deficits of Asian individuals. Furthermore, the intraobserver agreement and interobserver agreement suggest that the AMVDSS could be used by different evaluators or the same evaluator at different times and could achieve reliable assessments of midface volume deficits and volume changes after dermal filler injections. Additionally, the AMVDSS may be an objective assessment tool that clinicians could use to communicate effectively with Asian patients about midface rejuvenation.
